# Using systems thinking to generate novel research questions for the evaluation of sugar-sweetened beverage taxation policies

**DOI:** 10.1136/bmjgh-2023-012060

**Published:** 2023-10-09

**Authors:** Miriam Alvarado, Robert Marten, Leandro Garcia, Aku Kwamie, Martin White, Jean Adams

**Affiliations:** 1 MRC Epidemiology Unit, University of Cambridge, Cambridge, UK; 2 George Alleyne Chronic Disease Research Centre, The University of the West Indies, Bridgetown, Barbados; 3 Alliance For Health Policy and System Research, Geneva, Switzerland; 4 Centre for Public Health, Queen's University Belfast, Belfast, UK

**Keywords:** Health policy, Health systems evaluation, Nutrition, Prevention strategies

**Summary box**

- Sugar-sweetened beverage (SSB) taxes have been recommended by the WHO, introduced in over 85 countries, and assessed through an increasing number of evaluations.
- However, few studies have applied systems thinking to generate questions exploring the range of ways in which an SSB tax, and the system within which it is embedded, have adapted to one another over time.
- We demonstrate how adopting this perspective could advance the field. A systems thinking perspective focuses on how the intervention and system adapt to one another. Interventions are viewed as disruptions to a complex system in which the system may change how the intervention operates over time, and the system may coevolve to minimise or amplify disturbances caused by the intervention.
- As the literature on SSB taxes matures, there is a scope to employ a systems perspective to develop novel research questions, based on a deeper understanding of the system’s boundaries, its observed patterns over time, and its underpinning assumptions and structure.
- Addressing systems-informed questions could address gaps in the SSB literature, including: (1) considering intervention and system coevolution over time, (2) identifying further unanticipated consequences and (3) building deeper understanding by drawing on diverse types of studies.
- A systems perspective offers insights that can help to strengthen health-promoting impacts, minimise health-harming impacts, and support policy sustainability and adaptation over time.
- We have focused on SSB taxation, but other types of health policy evaluations, including other health taxes, would also benefit from the increased use of systems thinking-informed research questions.

## Introduction

Sugar-sweetened beverage (SSB) taxes have been recommended by the WHO,^1^ introduced in over 85 countries,^1^ and assessed through an increasing number of evaluations.^2^ Experimental studies have considered efficacy questions, showing that, in virtual supermarkets and selected vending machines, SSB taxes reduce SSB purchases.^3–5^ Observational studies have addressed effectiveness questions, demonstrating that national SSB taxes successfully reduce real-world SSB sales.^2^ Other studies have examined theory-based questions, for example, assessing whether taxes lead to larger changes over time, as predicted by rational addiction theory.^6 7^ However, few studies have applied systems thinking to generate questions exploring the range of ways in which an SSB tax, and the system within which it is embedded, have adapted to one another over time. We demonstrate how adopting this perspective could advance the field; while we focus on SSB taxes, these arguments are relevant to evaluations of health policies more broadly.

## The need for a systems research perspective

Interventions such as SSB taxes are introduced into underlying economic, health and social systems. Because these are complex adaptive systems, they demonstrate properties such as non-linearity, feedback, path dependence, self-organisation, emergence, tipping points and time lags.^8^ A systems thinking perspective focuses on how the intervention and system adapt to one another.^9–11^ Interventions are viewed as disruptions to a complex system^12^ in which the system may change how the intervention operates over time, and the system may coevolve to minimise or amplify disturbances caused by the intervention.

To contextualise this systems thinking-informed approach to evaluation and highlight distinctions from other, more frequently applied approaches, we summarise and build on the new UK Medical Research Council and UK National Institute for Health and Care Research Framework for the development and evaluation of complex interventions^9^ in table 1 with examples relevant to SSB taxation.

**Table 1:** The new UK MRC and NIHR guidance on developing and evaluating complex interventions: four research perspectives and their application to sugar-sweetened beverage taxation^9^

Type of research question	General research question	Example research question	SSB taxation examples	Function
Efficacy	To what extent does the intervention produce the intended outcomes in experimental or ideal settings?	Does introducing an SSB tax in an experimental virtual supermarket setting have an impact on SSB sales?	Waterlander *et al,*^3^ 2019 Seah *et al,*^4^ 2018 Bos *et al,*^5^ 2018	Identification and justification for promising policy interventions
Effectiveness	To what extent does the intervention produce the intended outcomes in real world settings?	To what extent does introducing a national SSB tax have an impact on SSB sales?	Andreyeva *et al,*^2^ 2022 (a review of effectiveness studies)	Assessment of intended impacts, quantification of effect size
Theory based	What works in which circumstances and how?	What types of SSB taxes reduce SSB sales in which settings (or for which subgroups), and how are these effects achieved?	Le Bodo *et al,*^30^ 2019 Forde *et al*,^31^ 2022	Exploration of mechanisms, role of context and potential to generalise to other settings, assessment of unintended consequences
Systems	How do the system and intervention adapt to one another (over time)?	How does the network of stakeholders adapt to a tax over time? How does the system within which a tax is introduced influence the tax itself over time (eg, changing design, rate, longevity)?	Urwannachotima *et al,*^25^ 2019	Explanation of how and why intervention and system coevolve in response to each other, identification of further unintended consequences

MRC, Medical Research Council; NIHR, National Institute for Health and Care Research; SSB, sugar-sweetened beverage.

As demonstrated in table 1, the kinds of questions that are associated with each approach vary substantially. While effectiveness questions have high policy relevance and are often the subject of numerous evaluations (and systematic reviews), we risk missing key insights if we do not diversify the types of questions that are posed in future evaluations. While insights developed from an effectiveness perspective may inform decisions to keep or scrap a policy based on whether it ‘works’, insights from a systems perspective may inform decisions about how to improve or adapt a policy in response to a changing and uncertain context.

## Addressing existing gaps in the SSB taxation literature

As the literature on SSB taxes matures, there is a scope to employ a systems perspective to develop novel research questions, based on a deeper understanding of the system’s boundaries, its observed patterns over time, and its underpinning assumptions and structure. Addressing systems-informed questions could address gaps in the SSB literature, including: (1) considering intervention and system coevolution over time, (2) identifying further unanticipated consequences and (3) building deeper understanding by drawing on diverse types of studies. We discuss each in more detail below and then discuss how to develop systems thinking-informed research questions.

### Addressing gap 1: intervention and system coevolution over time

Many SSB tax evaluations have considered factors leading to the introduction of a tax.^13–17^ However, SSB taxes may continue to be contested after introduction, with important consequences for health impacts. For example, promising efforts to increase the SSB tax rate in South Africa were stalled, while in the UK the SSB tax has faced repeated calls for repeal. In Barbados, an initial tax design has been progressively improved by first amending the definition of taxable products and later doubling the tax rate.^18^ A systems thinking approach encourages researchers to explicitly consider SSB taxation as an intervention that will likely evolve in response to the adaptive system into which it is introduced. This may entail, for example, posing questions about the interrelated factors that contribute to the strengthening or weakening of SSB tax policies over time (ie, ways in which the system may impact the tax). It may also involve developing questions around the ways in which an SSB tax may shift the underlying acceptability of other types of regulations to improve dietary public health (ie, ways in which the tax may change the system). By focusing attention on intervention-system evolution, a systems perspective encourages researchers to consider novel research questions.

### Addressing gap 2: identifying further unanticipated consequences

Systems thinking approaches can help researchers to hypothesise additional consequences of an SSB tax which would not have been evident by relying on an intervention-centred theory of change alone. When the underlying system is explicitly described, additional unintended effects may be imagined and subsequently assessed, enriching our understanding of ‘what happened’ following the introduction of an SSB tax. Hypothesised systemic effects can be identified in a range of ways (eg, through participatory system mapping approaches such as group model building.^19^ Engaging a wide array of system stakeholders can bring multiple and diverse perspectives to bear on the development of these shared hypotheses. Some unintended consequences may dampen the health impacts of a tax (eg, increased SSB marketing), while others may amplify health-promoting effects (eg, a decrease in the social acceptability of SSB consumption). In either case, having a clearer holistic understanding of what happens when a tax is introduced would enable guidance to be developed to amplify positive effects, mitigate negative ones and adapt the policy in response to system evolution.

### Addressing gap 3: building greater meaning across diverse types of studies

Qualitative methods are often used to consider how and why a tax was introduced; quantitative observational methods are often used to consider changes in key outcomes; and quantitative modelling is often used to predict long-term impacts. These different study designs shed light on particular aspects of SSB taxation but are often published individually and synthesised alongside similar studies, producing silos of results that focus on narrow questions using similar methods. This prevents researchers from producing insights that are qualitatively different from the sum of their parts. A more holistic, systems perspective may help us to pose compound, integrative questions.^20^ For example, we could ask: is a reinforcing feedback loop created if the introduction of a tax reduces the number of SSB consumers, and this reduction leads to an increase in the political acceptability of taxing SSBs further? Are there other balancing feedback loops which counter this effect? By posing questions which draw on multiple types of data simultaneously, a systems research perspective may create a meaningful way to integrate different kinds of evidence so as to develop broader insights.

## Developing systems thinking-informed research questions

While posing research questions from a systems perspective remains relatively rare in SSB tax evaluations, increasingly, guidance is available.^9 21–24^ For example, McGill *et al* describe a two-stage process for developing systems research questions as part of a process evaluation.^22^ The first phase involves the development of an understanding of the system (at or before the time when the intervention is introduced). The second phase involves hypothesising how the system undergoes changes following the introduction of the intervention, how the system may change the intervention, and how these dynamics evolve over time. The resulting hypotheses form the basis for research questions posed from a systems perspective.

As an applied example, Urwannachotima *et al* used group model building to develop systems research questions regarding a proposed SSB tax in Thailand.^25^ They conducted a series of workshops with stakeholders to develop causal loop diagrams (CLDs) linking SSB taxation and dental caries. Based on these CLDs, they hypothesised that an SSB tax alone would be insufficient to reduce dental caries because of increasing income/affordability, potential substitution to untaxed high sugar drinks and industry-led tax absorption in response to falling sales. These hypotheses can be reframed as a series of systems-informed research questions: Are incomes rising fast enough to offset tax-induced price changes in the cost of SSBs? Do consumers adapt to the introduction of a tax by substituting to other untaxed high-sugar drinks sold at coffee shops and markets (an important feature of the Thai context)? Do manufacturers adapt to tax-induced reductions in sales by increasingly absorbing the tax?

The process of developing systems-informed research questions can be facilitated by using CLDs or other systems science approaches, such as system dynamics modelling, which enable a description of an underlying system and simulation of the disruption caused by an intervention. The hypotheses and research questions developed using systems methods can then be used as the foundation for empirical evaluation. Notably, the subsequent empirical evaluations do not necessarily require systems science methods, but should draw on systems thinking concepts to ensure the continuity of a systems perspective throughout the evaluation.^26 27^

## Conclusion

Applying systems thinking to SSB taxes will help to address key gaps in the SSB tax evidence base and, importantly, take a longer-term and broader perspective on their impact. The answers to research questions posed from a systems perspective may be nuanced and differ from the kinds of results we are familiar with. However, these insights offer an opportunity to strengthen policies and associated health-promoting impacts, minimise health-harming impacts, and support policy sustainability and adaptation over time. We have focused on SSB taxation, but other types of health policy evaluations would also benefit from the increased use of systems thinking-informed research questions.^28 29^

## Data Availability

There are no data in this work.
